# Detection of the T790M mutation of *EGFR* in plasma of advanced non–small cell lung cancer patients with acquired resistance to tyrosine kinase inhibitors (West Japan oncology group 8014LTR study)

**DOI:** 10.18632/oncotarget.11303

**Published:** 2016-08-16

**Authors:** Takayuki Takahama, Kazuko Sakai, Masayuki Takeda, Koichi Azuma, Toyoaki Hida, Masataka Hirabayashi, Tetsuya Oguri, Hiroshi Tanaka, Noriyuki Ebi, Toshiyuki Sawa, Akihiro Bessho, Motoko Tachihara, Hiroaki Akamatsu, Shuji Bandoh, Daisuke Himeji, Tatsuo Ohira, Mototsugu Shimokawa, Yoichi Nakanishi, Kazuhiko Nakagawa, Kazuto Nishio

**Affiliations:** ^1^ Department of Medical Oncology, Kinki University Faculty of Medicine, Osaka, Japan; ^2^ Department of Genome Biology, Kinki University Faculty of Medicine, Osaka, Japan; ^3^ Department of Internal Medicine, Division of Respirology, Neurology, and Rheumatology, Kurume University School of Medicine, Kurume, Fukuoka, Japan; ^4^ Department of Thoracic Oncology, Aichi Cancer Center, Nagoya, Japan; ^5^ Department of Respiratory Medicine, Hyogo Prefectural Amagasaki General Medical Center, Hyogo, Japan; ^6^ Department of Medical Oncology and Immunology, Nagoya City University Graduate School of Medical Sciences, Nagoya, Japan; ^7^ Department of Internal Medicine, Niigata Cancer Center Hospital, Niigata, Japan; ^8^ Department of Respiratory Oncology, Iizuka Hospital, Fukuoka, Japan; ^9^ Department of Respiratory Medicine and Oncology, Gifu Municipal Hospital, Gifu, Japan; ^10^ Department of Pulmonary Medicine, Japanese Red Cross Okayama Hospital, Okayama, Japan; ^11^ Division of Respiratory Medicine, Department of Internal Medicine, Kobe University Graduate School of Medicine, Hyogo, Japan; ^12^ Third Department of Internal Medicine, Wakayama Medical University, Wakayama, Japan; ^13^ Division of Hematology, Rheumatology, and Respiratory Medicine, Department of Internal Medicine, Faculty of Medicine, Kagawa University, Kagawa, Japan; ^14^ Department of Internal Medicine, Miyazaki Prefectural Miyazaki Hospital, Miyazaki, Japan; ^15^ Department of Surgery, Tokyo Medical University, Tokyo, Japan; ^16^ Department of Cancer Information Research, National Kyushu Cancer Center, Fukuoka, Japan; ^17^ Research Institute for Diseases of the Chest, Graduate School of Medical Sciences, Kyushu University, Fukuoka, Japan

**Keywords:** non–small cell lung cancer, epidermal growth factor receptor (EGFR), mutation, T790M, cell-free DNA

## Abstract

**Introduction:**

Next-generation epidermal growth factor receptor (EGFR) tyrosine kinase inhibitors (TKIs) have been developed to overcome resistance to earlier generations of such drugs mediated by a secondary T790M mutation of *EGFR*, but the performance of a second tumor biopsy to assess T790M mutation status can be problematic.

**Methods:**

We developed and evaluated liquid biopsy assays for detection of TKI-sensitizing and T790M mutations of *EGFR* by droplet digital PCR (ddPCR) in *EGFR* mutation–positive non–small cell lung cancer (NSCLC) patients with acquired EGFR-TKI resistance.

**Results:**

A total of 260 patients was enrolled between November 2014 and March 2015 at 29 centers for this West Japan Oncology Group (WJOG 8014LTR) study. Plasma specimens from all subjects as well as tumor tissue or malignant pleural effusion or ascites fluid from 41 patients were collected after the development of EGFR-TKI resistance. All plasma samples were genotyped successfully and the results were reported to physicians within 14 days. TKI-sensitizing and T790M mutations were detected in plasma of 120 (46.2%) and 75 (28.8%) patients, respectively. T790M was detected in 56.7% of patients with plasma positive for TKI-sensitizing mutations. For the 41 patients with paired samples obtained after acquisition of EGFR-TKI resistance, the concordance for mutation detection by ddPCR in plasma compared with tumor tissue or malignant fluid specimens was 78.0% for TKI-sensitizing mutations and 65.9% for T790M.

**Conclusions:**

Noninvasive genotyping by ddPCR with cell-free DNA extracted from plasma is a promising approach to the detection of gene mutations during targeted treatment.

## INTRODUCTION

Most non–small cell lung cancer (NSCLC) tumors develop resistance to epidermal growth factor receptor (EGFR) tyrosine kinase inhibitors (TKIs) within 10 to 16 months after the initiation of EGFR-TKI therapy [1–6]. Several mechanisms of such acquired resistance—including a secondary T790M mutation in exon 20 of *EGFR*, *MET* amplification, overexpression of hepatocyte growth factor, and activation of the insulin-like growth factor 1 receptor—have been identified. The T790M mutation of *EGFR* is the most common cause of acquired resistance to EGFR-TKIs, being found in up to 50% of patients treated with these drugs [7–10], and several next-generation EGFR-TKIs, such as CO-1686 and AZD9291 (irreversible T790M mutant–specific EGFR-TKI with little inhibitory activity for wild-type EGFR), have been developed to overcome such resistance [11–13]. However, the performance of a second biopsy to assess T790M mutation status can be problematic depending on the size and location of the tumor tissue, possibly requiring invasive procedures such as mediastinoscopy or video-assisted thoracoscopy.

Liquid biopsy, a noninvasive means to detect cancer cell DNA in blood, has the potential to allow detection of cancer, measurement of tumor burden, and evaluation of drug sensitivity or resistance. In the present study, we prospectively examined whether droplet digital polymerase chain reaction (ddPCR) analysis of cell-free DNA (cfDNA) might allow highly specific and quantitative assessment of TKI-sensitizing and T790M resistance mutations of *EGFR* in patients with advanced NSCLC who acquire resistance to EGFR-TKI therapy.

## RESULTS

### Cutoff values for prescreening

To optimize the specificity of our *EGFR* genotyping assays, we determined the cutoff values for plasma cfDNA, pleural effusion or ascites fluid, and formalin-fixed, paraffin-embedded (FFPE) specimens with plasma cfDNA derived from 10 healthy volunteers, normal genomic DNA (Promega, Madison, WI), and *EGFR* mutation–negative FFPE samples. No background noise (0 copies per reaction) was detected for assay of *EGFR* harboring T790M, L858R, E746-A750del, L861Q, or G719X mutations with plasma cfDNA derived from each of the 10 healthy volunteers or with the normal genomic DNA. The cutoff value for each *EGFR* mutation was therefore set at 3 copies per reaction (20 μL), or 0.15 copies/μL, for plasma cfDNA. The DNA extracted from pleural effusion or ascites fluid was of high molecular weight similar to that isolated from blood plasma. The same cutoff value was therefore selected for these specimens. DNA extracted from FFPE specimens is usually degraded, and 21 FFPE samples of *EGFR* mutation–negative NSCLC were used to assign cutoff values. The mean ± SD values for T790M, L858R, E746-A750del, L861Q, and G719X mutant copy number in these 21 samples were calculated, and the higher value of the mean + 3SD copy number or 3 copies per reaction was chosen as the cutoff for each mutation, consistent with the approach adopted in a previous study [14]. The cutoff values were thus set at 1.11 copies/μL for T790M, 0.2 copies/μL for L858R, 0.3 copies/μL for E746-A750del, 0.15 copies/μL for L861Q, and 1.8 copies/μL for G719X.

### Patient characteristics

We recruited 260 patients with *EGFR* mutation–positive NSCLC and acquired resistance to EGFR-TKIs from 29 institutions in Japan between 4 November 2014 and 13 March 2015 (Table 1). The subjects included 182 (70.0%) women and 186 (71.5%) never-smokers, with an overall median age of 68 years (range, 36 to 90). Most patients had disease of stage IIIb or IV at diagnosis (78.8%) and an Eastern Cooperative Oncology Group (ECOG) performance status of 0 to 2 (95.8%), and 191 (73.5%) received EGFR-TKI treatment as first-line therapy. With regard to the type of *EGFR* mutation identified by commercial assays with diagnostic FFPE samples, 127 (48.8%) patients had a deletion in exon 19, 122 (46.9%) had a missense mutation in exon 21 (L858R or L861Q), and 4 (1.5%) had a G719X mutation in exon 18. Most patients (78.8%) were treated with gefitinib as the first EGFR-TKI, and most plasma specimens (58.8%) were collected from patients treated with an EGFR-TKI as the immediate prior therapy.

**Table 1:** Characteristics of *EGFR* mutation–positive NSCLC patients with acquired resistance to EGFR-TKIs (*n* = 260)

	No. of patients (%)	Detection of TKI-sensitizing mutations in plasma cfDNA (%)	*P* (χ^2^) for sensitizing mutations	Detection of T790M in plasma cfDNA (%)	*P* (χ^2^) for T790M
Total	260 (100)	120 (46.2)		75 (28.8)	
[Median age (range) in years	68 (36–90)]				
Sex					
Male	78 (30.0)	40 (33.3)	0.5139	27 (36.0)	0.3262
Female	182 (70.0)	80 (66.7)		48 (64.0)	
Smoking history					
Never-smoker	186 (71.5)	82 (68.3)	0.7807	52 (69.3)	0.6242
Smoker	71 (27.3)	36 (30.0)		21 (28.0)	
Unknown	3 (1.2)	2 (1.7)		2 (2.7)	
*EGFR* mutation status at diagnosis					
Exon 19 deletion	127 (48.8)	59 (49.2)	0.8839	48 (64.0)	0.1284
L858R or L861Q	122 (46.9)	57 (47.5)		24 (32.0)	
With T790M^1^	6 (2.3)	2 (1.7)		2 (2.7)	
Other	5 (1.9)	2 (1.7)		1 (1.3)	
ECOG performance status					
0–2	249 (95.8)	114 (95.0)	0.831	69 (92.0)	0.3614
3–4	8 (3.1)	5 (4.2)		5 (6.7)	
Missing	3 (1.2)	1 (0.8)		1 (1.3)	
Disease stage					
IIIb/IV/inoperable	205 (78.8)	103 (85.8)	0.122	60 (80.0)	0.8735
Postoperative recurrence	55 (21.2)	17 (14.2)		15 (20.0)	
No. of previous cytotoxic chemotherapies					
0	142 (54.6)	46 (38.3)	0.004	25 (33.3)	0.0016
≥1	118 (45.4)	74 (61.7)		50 (66.7)	
Immediate prior treatment					
EGFR-TKI	153 (58.8)	74 (61.7)	0.7758	47 (62.7)	0.8203
Chemotherapy	104 (40.0)	44 (36.7)		27 (36.0)	
Other	3 (1.2)	2 (1.7)		1 (1.3)	
First EGFR-TKI					
Gefitinib	205 (78.8)	93 (77.5)	0.8711	56 (74.7))	0.1357
Erlotinib	47 (18.1)	24 (20.0)		19 (25.3)	
Afatinib	8 (3.1)	3 (2.5)		0 (0.0)	

1 T790M as well as an exon 19 deletion, L858R, or L861Q.

### Feasibility

DNA was extracted for ddPCR analysis from plasma specimens collected from *EGFR* mutation–positive NSCLC patients after the acquisition of resistance to EGFR-TKIs (*n* = 260). The median plasma specimen volume was 2.20 mL (range, 1.10–3.60), and the median amount of amplifiable DNA extracted from plasma was 4014.1 copies per sample (range, 611.5–162, 211.1), suggesting that plasma specimens were successfully processed for DNA extraction. Moreover, the success rate for each genotyping assay was 100%, and all genotyping results were reported to each physician within 14 days. Patients whose plasma tested positive for an EGFR-TKI–sensitizing *EGFR* mutation had a significantly higher DNA copy number than did those who tested negative (median of 4592.2 versus 3530.2; *P* = 0.035). The median DNA copy number for patients whose plasma tested positive for the T790M mutation was also higher than that for those who tested negative (4783.0 versus 3709.3, *P* = 0.127), although this difference was not significant.

### Relations between detection of TKI-sensitizing or T790M mutations of *EGFR* in plasma and clinicopathologic characteristics

Among the 260 plasma specimens obtained from patients after the development of acquired resistance to EGFR-TKIs, a TKI-sensitizing mutation was detected in 120 (46.2%) samples and a T790M mutation in 75 (28.8%) (Table 1). A T790M mutation was detected in 68 (56.7%) of the 120 patients whose plasma tested positive for a TKI-sensitizing mutation, with the other 7 plasma specimens positive for T790M testing negative for a TKI-sensitizing mutation.

We examined the relations of clinicopathologic characteristics to the detection rates for TKI-sensitizing and T790M mutations in plasma (Table 1). The detection rates for TKI-sensitizing or T790M mutations in plasma were not significantly related to sex, smoking status, *EGFR* mutation status at diagnosis, ECOG performance status, tumor stage, immediate prior treatment regimen, or type of EGFR-TKI first administered. However, they did show a significant association with the number of previous cytotoxic chemotherapy regimens, being higher in patients treated with one or more such regimens than in those not so treated (61.7% versus 38.3% for TKI-sensitizing mutations, *P* = 0.004; 66.7% versus 33.3% for T790M, *P* = 0.0016).

### Detection of TKI-sensitizing or T790M mutations of *EGFR* in second biopsy specimens

Tumor tissue specimens were obtained at secondary biopsy from 18 patients and malignant fluid (pleural effusion or ascites) was collected from 23 patients after the development of acquired resistance to EGFR-TKIs (Table 2). The ddPCR assays detected a TKI-sensitizing mutation in 33 (80.5%) and T790M in 31 (75.6%) of these 41 specimens. Given that the study recruited *EGFR* mutation–positive NSCLC patients, these results indicated that ddPCR could not detect ~20% of such mutations identified by commercial assays. We next examined the concordance for *EGFR* mutation detection according to mutation type between specimens obtained for diagnosis and those obtained after the development of EGFR-TKI resistance (Supplementary Table S1). Whereas 14 (93.3%) of the 15 patients positive for the L858R mutation at diagnosis were also positive for this mutation by ddPCR, only 19 (73.1%) of the 26 patients positive for exon 19 deletions at diagnosis were similarly positive by ddPCR.

**Table 2:** Detection of TKI-sensitizing and T790M mutations of *EGFR* in tumor tissue or malignant fluid specimens obtained after the development of EGFR-TKI resistance (*n* = 41)

Sample	Sensitizing mutation	T790M
Malignant fluid (pleural effusion or ascites) (*n* = 23)	19/23 (82.6%)	20/23 (87.0%)
Lung or lymph node (*n* = 18)	14/18 (77.8%)	11/18 (61.1%)
Total (*n* = 41)	33/41 (80.5%)	31/41 (75.6%)

### Concordance of *EGFR* mutation status between plasma and tumor samples

Among the 41 patients with tumor tissue or malignant fluid specimens collected after the acquisition of EGFR-TKI resistance, TKI-sensitizing mutations were detected in 19 (82.6%) of the 23 fluid samples and in 14 (77.8%) of the 18 tumor tissue samples obtained at rebiopsy, whereas the corresponding values for T790M were 20 (87.0%) out of 23 and 11 (61.1%) out of 18, respectively (Table 2). For these same 41 patients with paired plasma and either tumor tissue or malignant fluid samples obtained after the development of EGFR-TKI resistance, the concordance for mutation detection by ddPCR in plasma relative to that in the paired samples was 78.0% (32/41) (with a sensitivity of 75.8% and specificity of 87.5%) for TKI-sensitizing mutations and 65.9% (27/41) (with a sensitivity of 64.5% and specificity of 70.0%) for T790M (Table 3).

**Table 3:** Correlation for detection of TKI-sensitizing or T790M mutations of *EGFR* between plasma and either tumor tissue or malignant fluid (rebiopsy) specimens obtained after the development of EGFR-TKI resistance (*n* = 41)

		Plasma				
		Sensitizing mutation (+)	Sensitizing mutation (−)		T790M (+)	T790M (−)
Rebiopsy specimens	Sensitizing mutation (+)	25	8	T790M (+)	20	11
	Sensitizing mutation (−)	1	7	T790M (−)	3	7

## DISCUSSION

We have here performed a prospective evaluation of ddPCR for the sensitive and quantitative analysis of the T790M mutation of *EGFR* in plasma specimens from patients with *EGFR* mutation–positive NSCLC treated with EGFR-TKIs. All 260 plasma samples were successfully processed for DNA extraction, genotyping, and reporting of the results to the treating physician within 14 days, giving an overall success rate of 100%. Our results thus suggest that ddPCR is a suitable method for detection of the T790M mutation of *EGFR* in plasma samples.

We detected the T790M mutation of *EGFR* at a frequency of 28.8% in plasma cfDNA of EGFR-TKI–treated patients, a value lower than that reported in a previous study (43%) investigating the digital PCR–based quantification of T790M in plasma cfDNA of patients with advanced NSCLC treated with EGFR-TKIs [15]. This apparent discrepancy might be due to a difference in the timing of plasma collection between the two studies. Our study had no restriction regarding previous treatment including cytotoxic chemotherapy, and we found that the T790M detection rate was significantly higher in patients who received one or more cytotoxic chemotherapy regimens than in those who did not receive such therapy. In contrast to our study, the former study restricted collection of plasma specimens to the time of documented disease progression during EGFR-TKI treatment and before the onset of a subsequent treatment. Furthermore, most patients (73.5%) in the present study received EGFR-TKIs as a first-line treatment, whereas most patients (69.6%) in the former study received these drugs as a second-line or later treatment. A previous study of T790M in patients with *EGFR* mutation–positive NSCLC before treatment detected this mutation more frequently in individuals with larger tumors [16]. These observations suggest that larger tumors and more heavily treated tumors might contain a greater number of rare T790M subclones. The T790M mutation might therefore be detected at a higher frequency in plasma samples obtained from patients after the development of progressive disease during late-line EGFR-TKI treatment.

The frequency of the T790M mutation in plasma specimens that tested positive for a TKI-sensitizing mutation was 56.7%, a value similar to that (~50%) previously determined for the detection of T790M in tissue samples of *EGFR* mutation–positive tumors with acquired resistance to EGFR-TKIs [7–10]. The concordance for T790M detection by digital PCR between tumor tissue and plasma DNA obtained after the development of resistance to EGFR-TKI therapy was previously found to be 64% [15]. Consistent with this previous finding, we found that the concordance for T790M mutation detection by ddPCR with paired plasma and either tumor tissue or malignant fluid samples obtained after the development of EGFR-TKI resistance was 65.9%. Together, these previous and present results suggest that digital PCR analysis of plasma cfDNA is a promising strategy for detection of the T790M mutation in patients whose plasma also tests positive for a TKI-sensitizing mutation. Moreover, 28.1% of patients (73/260) enrolled in our study had only extrathoracic metastatic lesions including those in the brain, bone, and adrenal gland. TKI-sensitizing and T790M mutations were detected in plasma of 45 (61.6%) and 34 (46.6%) of these 73 patients, respectively, suggesting that noninvasive ddPCR analysis is able to detect *EGFR* mutations for patients whose tumors are difficult to access for rebiopsy.

With regard to limitations of our study, the accuracy of ddPCR for detection of exon 19 deletions of *EGFR* may be less than that of commercial assays such as Scorpion ARMS and the Cobas *EGFR* Mutation Test (Supplementary Table S1), given that ddPCR is not able to detect all types of in-frame deletion. In addition, some discordance for detection of T790M between plasma and either tumor tissue or malignant fluid samples obtained after the development of resistance to EGFR-TKIs was observed and found to be largely due to false negative results with the plasma samples (Table 3). Given that the T790M mutation was detected by ddPCR at a high frequency in malignant fluid samples, such samples might be more suitable than plasma for such analysis when available. Third-generation EGFR-TKIs such as AZD9291 (osimertinib) had not been approved by the Japanese Drug Regulatory Agency during the study period, and so we were not able to determine the clinical outcome of such treated patients with a T790M mutation detected in plasma cfDNA. We have therefore initiated a prospective phase II study (UMIN 000022076) to assess the clinical efficacy of AZD9291 in patients who develop T790M-mediated resistance to first-generation EGFR-TKIs as detected by ddPCR analysis of plasma cfDNA.

In conclusion, noninvasive genotyping by ddPCR analysis of plasma cfDNA is a promising approach for the detection of gene mutations that arise during treatment with EGFR-TKIs or other targeted drugs, which is an important aspect of the optimization of personalized therapy.

## MATERIALS AND METHODS

### Patient selection

Patients were prospectively enrolled in this West Japan Oncology Group study (WJOG 8014LTR) according to the following inclusion criteria: (i) the presence of *EGFR* mutation–positive NSCLC identified by commercial assays such as Scorpion ARMS and the Cobas *EGFR* Mutation Test, and (ii) documented disease progression based on RECIST or worsening of lung cancer symptoms during EGFR-TKI treatment. Patients who manifested isolated progression in the central nervous system or bone were allowed to enroll, whereas those who permanently discontinued treatment with any EGFR-TKI because of an adverse event without disease progression were excluded. No restrictions related to previous treatment (including cytotoxic chemotherapy), performance status, or other factors were imposed. All patients provided written informed consent to participation in the study, including the collection of tumor and plasma specimens for analysis. The study was approved by the independent ethics committee at each institution, was performed in accordance with the Declaration of Helsinki, and is registered with UMIN (University Hospital Medical Information Network in Japan) under accession number 000015461.

### Specimen collection

Whole blood (7 mL) was collected from the study subjects after they had developed resistance to EGFR-TKIs. The EDTA-treated blood was centrifuged at 1400 × *g* for 10 min, and the plasma supernatant was stored at −80°C until analysis. Matched tumor tissue specimens or malignant pleural effusion or ascites fluid were also collected if possible. Tumor tissue was stored as FFPE samples, and malignant pleural effusion or ascites fluid was centrifuged at each institution and the resulting supernatants were stored at −80°C. Plasma, pleural effusion, and ascites DNA was purified with the use of a QIAamp Circulating Nucleic Acid Kit (Qiagen, Valencia, CA), whereas tumor tissue DNA was purified with a QIAamp DNA Micro Kit (Qiagen). The copy number for extracted cfDNA was determined with an RNaseP Copy Number Assay (Life Technologies, Carlsbad, CA). The extracted DNA was stored at 4°C until analysis.

### Droplet digital PCR assays

Mutant allele frequency for *EGFR* was measured with the use of the QX100 Droplet Digital PCR System (Bio-Rad, Hercules, CA). The primers and probes for detection of the E746-A750 deletion, L858R, L861Q, and T790M were obtained from Bio-Rad. Primers and probes for G719X were designed to detect G719S, G719C, and G719A: G719X forward primer, 5′-TGAAGGAAACTGAATTCAAAA-3′; G719X reverse primer, 5′-CTTACCTTATACACCGTGC-3′; G719S probe, 5′-/56-FAM/AGTGCTGTC/ZEN/CTCCGG/3IABkFQ/-3′; G719C probe, 5′-/56-FAM/AAAGTGCTG/ZEN/TGCTCCG/3IABkFQ/-3′; G719A probe, 5′-/56-FAM/TGCTGGCCT/ZEN/CCGG/3IABkFQ/-3′; and G719G-WT probe, 5′-/5HEX/AGTGCTGGG/ZEN/CTCCG/3IABkFQ/-3′. The cycling conditions for the PCR reaction included an initial incubation at 95°C for 10 min, 40 cycles of 94°C for 30 s and 55°C for 60 s, and enzyme inactivation at 98°C for 10 min. After thermal cycling, the plates were transferred to a Droplet reader (Bio-Rad). The digital PCR data were analyzed with the Quanta Soft analytical software package (Bio-Rad).

### Statistical analysis

The primary end point of the study was to estimate the overall success rate and its 95% confidence interval (CI) for genotyping by ddPCR and for informing each physician of the genotyping results within 14 days, with the study being considered feasible if the upper limit of the 95% CI is >90%. The planned enrollment was 155 patients. For all assays, this sample size was considered the minimum required to estimate a 95% CI for the true overall success rate within a width of ±0.05, with the success rate expected to be ≥90% taking into account the anticipated frequencies of technical failure and specimens with degraded or insufficient DNA. The relations between the presence of TKI-sensitizing or T790M mutations and patient characteristics were evaluated with the chi-square (χ^2^) test. A *P* value of <0.05 was considered statistically significant. All statistical analysis was performed with the use of Prism software (GraphPad Software).

## SUPPLEMENTARY FIGURES AND TABLES



## References

[1] Maemondo M, Inoue A, Kobayashi K, Sugawara S, Oizumi S, Isobe H, Gemma A, Harada M, Yoshizawa H, Kinoshita I, Fujita Y, Okinaga S, Hirano H, Yoshimori K, Harada T, Ogura T (2010). Gefitinib or chemotherapy for non-small-cell lung cancer with mutated EGFR. N Engl J Med.

[2] Mitsudomi T, Morita S, Yatabe Y, Negoro S, Okamoto I, Tsurutani J, Seto T, Satouchi M, Tada H, Hirashima T, Asami K, Katakami N, Takada M, Yoshioka H, Shibata K, Kudoh S (2010). Gefitinib versus cisplatin plus docetaxel in patients with non-small-cell lung cancer harbouring mutations of the epidermal growth factor receptor (WJTOG3405): an open label, randomised phase 3 trial. Lancet Oncol.

[3] Zhou C, Wu YL, Chen G, Feng J, Liu XQ, Wang C, Zhang S, Wang J, Zhou S, Ren S, Lu S, Zhang L, Hu C, Hu C, Luo Y, Chen L (2011). Erlotinib versus chemotherapy as first-line treatment for patients with advanced EGFR mutation-positive non-small-cell lung cancer (OPTIMAL, CTONG-0802): a multicentre, open-label, randomised, phase 3 study. Lancet Oncol.

[4] Wu YL, Zhou C, Hu CP, Feng J, Lu S, Huang Y, Li W, Hou M, Shi JH, Lee KY, Xu CR, Massey D, Kim M, Shi Y, Geater SL (2014). Afatinib versus cisplatin plus gemcitabine for first-line treatment of Asian patients with advanced non-small-cell lung cancer harbouring EGFR mutations (LUX-Lung 6): an open-label, randomised phase 3 trial. Lancet Oncol.

[5] Sequist LV, Yang JC, Yamamoto N, O'Byrne K, Hirsh V, Mok T, Geater SL, Orlov S, Tsai CM, Boyer M, Su WC, Bennouna J, Kato T, Gorbunova V, Lee KH, Shah R (2013). Phase III study of afatinib or cisplatin plus pemetrexed in patients with metastatic lung adenocarcinoma with EGFR mutations. J Clin Oncol.

[6] Rosell R, Carcereny E, Gervais R, Vergnenegre A, Massuti B, Felip E, Palmero R, Garcia-Gomez R, Pallares C, Sanchez JM, Porta R, Cobo M, Garrido P, Longo F, Moran T, Insa A (2012). Erlotinib versus standard chemotherapy as first-line treatment for European patients with advanced EGFR mutation-positive non-small-cell lung cancer (EURTAC): a multicentre, open-label, randomised phase 3 trial. Lancet Oncol.

[7] Sequist LV, Waltman BA, Dias-Santagata D, Digumarthy S, Turke AB, Fidias P, Bergethon K, Shaw AT, Gettinger S, Cosper AK, Akhavanfard S, Heist RS, Temel J, Christensen JG, Wain JC, Lynch TJ (2011). Genotypic and histological evolution of lung cancers acquiring resistance to EGFR inhibitors. Sci Transl Med.

[8] Ohashi K, Maruvka YE, Michor F, Pao W (2013). Epidermal growth factor receptor tyrosine kinase inhibitor-resistant disease. J Clin Oncol.

[9] Kobayashi S, Boggon TJ, Dayaram T, Janne PA, Kocher O, Meyerson M, Johnson BE, Eck MJ, Tenen DG, Halmos B (2005). EGFR mutation and resistance of non-small-cell lung cancer to gefitinib. N Engl J Med.

[10] Yano S, Yamada T, Takeuchi S, Tachibana K, Minami Y, Yatabe Y, Mitsudomi T, Tanaka H, Kimura T, Kudoh S, Nokihara H, Ohe Y, Yokota J, Uramoto H, Yasumoto K, Kiura K (2011). Hepatocyte growth factor expression in EGFR mutant lung cancer with intrinsic and acquired resistance to tyrosine kinase inhibitors in a Japanese cohort. J Thorac Oncol.

[11] Janne PA, Yang JC, Kim DW, Planchard D, Ohe Y, Ramalingam SS, Ahn MJ, Kim SW, Su WC, Horn L, Haggstrom D, Felip E, Kim JH, Frewer P, Cantarini M, Brown KH (2015). AZD9291 in EGFR inhibitor-resistant non-small-cell lung cancer. N Engl J Med.

[12] Sequist LV, Soria JC, Goldman JW, Wakelee HA, Gadgeel SM, Varga A, Papadimitrakopoulou V, Solomon BJ, Oxnard GR, Dziadziuszko R, Aisner DL, Doebele RC, Galasso C, Garon EB, Heist RS, Logan J (2015). Rociletinib in EGFR-mutated non-small-cell lung cancer. N Engl J Med.

[13] Cross DA, Ashton SE, Ghiorghiu S, Eberlein C, Nebhan CA, Spitzler PJ, Orme JP, Finlay MR, Ward RA, Mellor MJ, Hughes G, Rahi A, Jacobs VN, Red Brewer M, Ichihara E, Sun J (2014). AZD9291, an irreversible EGFR TKI, overcomes T790M-mediated resistance to EGFR inhibitors in lung cancer. Cancer Discov.

[14] Sakai K, Tsurutani J, Yamanaka T, Yoneshige A, Ito A, Togashi Y, De Velasco MA, Terashima M, Fujita Y, Tomida S, Tamura T, Nakagawa K, Nishio K (2015). Extended RAS and BRAF Mutation Analysis Using Next-Generation Sequencing. PLoS One.

[15] Wang Z, Chen R, Wang S, Zhong J, Wu M, Zhao J, Duan J, Zhuo M, An T, Wang Y, Bai H, Wang J (2014). Quantification and dynamic monitoring of EGFR T790M in plasma cell-free DNA by digital PCR for prognosis of EGFR-TKI treatment in advanced NSCLC. PLoS One.

[16] Watanabe M, Kawaguchi T, Isa SI, Ando M, Tamiya A, Kubo A, Saka H, Takeo S, Adachi H, Tagawa T, Kakegawa S, Yamashita M, Kataoka K, Ichinose Y, Takeuchi Y, Sakamoto K (2015). Ultra-Sensitive Detection of the Pretreatment EGFR T790M Mutation in Non-Small Cell Lung Cancer Patients with an EGFR-Activating Mutation Using Droplet Digital PCR. Clin Cancer Res.

