# Supplementary material for: Detection of the T790M mutation of EGFR in plasma of advanced non–small cell lung cancer patients with acquired resistance to tyrosine kinase inhibitors (West Japan oncology group 8014LTR study)
Source: Oncotarget. 2016 Aug 16;7(36):58492–9. doi: 10.18632/oncotarget.11303 (PMC5295446; doi:10.18632/oncotarget.11303)
Supplement: Supplementary file 1 [file oncotarget-07-58492-s001.pdf]

Detection of the T790M mutation of *EGFR* in plasma of advanced non–small cell lung cancer patients with acquired resistance to tyrosine kinase inhibitors (West Japan oncology group 8014LTR study)

SUPPLEMENTARY TABLE

Supplementary Table S1: Detection of TKI-sensitizing and T790M mutations of *EGFR* both in diagnostic specimens and in either tumor tissue or malignant fluid (rebiopsy) specimens obtained after the development of EGFR-TKI resistance (n = 41)

EGFR mutation status	Diagnostic specimens (commercial assays)	Rebiopsy specimens (ddPCR)
L858R	15	14
Exon 19 deletion	26	19
T790M	0	31
